# Correlation Between the Social Network Structure and Well-Being of Health Care Workers in Intensive Care Units: Prospective Observational Study

**DOI:** 10.2196/50148

**Published:** 2023-11-29

**Authors:** Ryo Esumi, Asami Ito-Masui, Eiji Kawamoto, Mami Ito, Tomoyo Hayashi, Toru Shinkai, Atsuya Hane, Fumito Okuno, Eun Jeong Park, Ryuji Kaku, Motomu Shimaoka

**Affiliations:** 1 Department of Molecular Pathobiology and Cell Adhesion Biology Mie University Graduate School of Medicine Tsu Japan; 2 Department of Emergency Medicine National Hospital Organization Mie Chuo Medical Center Tsu Japan; 3 Department of Emergency and Disaster Medicine Mie University Hospital Tsu Japan; 4 Department of Anesthesiology Mie University Hospital Tsu Japan; 5 Department of Nursing Mie University Hospital Tsu Japan

**Keywords:** social network analysis, Center for Epidemiological Studies–Depression, CES-D, distributed leadership, intensive care unit, wearable sensor, face-to-face interaction

## Abstract

**Background:**

Effective communication strategies are becoming increasingly important in intensive care units (ICUs) where patients at high risk are treated. Distributed leadership promotes effective communication among health care professionals (HCPs). Moreover, beyond facilitating patient care, it may improve well-being among HCPs by fostering teamwork. However, the impact of distributed leadership on the communication structure and well-being of HCPs remains unclear.

**Objective:**

We performed a social network analysis (SNA) to assess the characteristics of each HCP in the network, identify the number of HCP connections, analyze 4 centralities that can measure an HCP’s importance, and evaluate the impact of distributed leadership structure on the well-being and communication structure of the medical staff.

**Methods:**

Wearable sensors were used to obtain face-to-face interaction data from the ICU medical staff at Mie University Hospital, Japan. Participants wore a badge on the front of their clothing during working hours to measure the total frequency of face-to-face interactions. We collected data about the well-being of medical staff using the Center for Epidemiological Studies–Depression (CES-D) questionnaire and measured 4 centralities using SNA analysis. A CES-D questionnaire was administered during the study to measure the well-being of the HCPs.

**Results:**

Overall, 247 ICU workers participated in this clinical study for 4 weeks yearly in February 2016, 2017, and 2018. The distributed leadership structure was established within the ICU in 2017 and 2018. We compared these results with those of the traditional leadership structure used in 2016. Most face-to-face interactions in the ICU were among nurses or between nurses and other professionals. In 2016, overall, 10 nurses could perform leadership tasks, which significantly increased to 24 in 2017 (*P*=.046) and 20 in 2018 (*P*=.046). Considering the increased number of nurses who could perform leadership duties and the collaboration created within the organization, SNA in 2018 showed that the betweenness (*P*=.001), degree (*P*<.001), and closeness (*P*<.001) centralities significantly increased compared with those in 2016. However, the eigenvector centrality significantly decreased in 2018 compared with that in 2016 (*P*=.01). The CES-D scores in 2018 also significantly decreased compared with those in 2016 (*P*=.01). The betweenness (*r*=0.269; *P*=.02), degree (*r*=0.262; *P*=.03), and eigenvector (*r*=0.261; *P*=.03) centralities and CES-D scores were positively correlated in 2016, whereas the closeness centrality and CES-D scores were negatively correlated (*r*=−0.318; *P*=.01). In 2018, the degree (*r*=−0.280; *P*=.01) and eigenvector (*r*=−0.284; *P*=.01) centralities were negatively correlated with CES-D scores.

**Conclusions:**

Face-to-face interactions of HCPs in the ICU were measured using wearable sensors, and nurses were found to be centrally located. However, the introduction of distributed leadership created collaboration and informal leadership in the organization, altering the social network structure of HCPs and increasing organizational well-being.

**Trial Registration:**

University Hospital Medical Information Network (UMIN) UMIN000037046; https://center6.umin.ac.jp/cgi-open-bin/icdr_e/ctr_view.cgi?recptno=R000042211

## Introduction

### Background

Effective communication in the intensive care unit (ICU) is critical for medical safety. However, poor communication in health care can lead to poor understanding among health care professionals, resulting in medication errors and jeopardizing patient safety. According to the Joint Commission, which is a US health care evaluation agency, <60% of the adverse events in hospitals in the United States are associated with poor communication [1]. In addition, poor communication among health care professionals can result in surgical complications and death [2,3]. Effective communication strategies, characterized by clarity, accuracy, and urgency, are becoming increasingly important in wards where patients at high risk are treated, such as ICUs and emergency departments. Therefore, the details of communication structures among health care professionals in the ICU, particularly the connections among staff, are important for patient safety [4].

Leadership structures have received attention as one of the key factors influencing the communication structure of health care professionals. Leadership in health care is generally defined as “the process by which an individual influences a group to achieve a common goal” [5]. Leadership structures can be categorized into two main categories as follows: (1) traditional leadership structures, where a single health care professional oversees the chain of command, and (2) distributed leadership structures, where multiple health care professionals form a team to perform medical tasks. Traditional leadership structure implies that a single leader makes decisions for the medical team and directs the organization or group; however, it is difficult for a single medical professional to make all important decisions in today’s diversified medical care. In contrast, a distributed leadership structure comprises ≥2 people (not necessarily all members of an organization) [6]. Although these 2 structures are frequently considered incompatible, they complement each other [5]. However, a distributed leadership structure would likely work to the advantage of managing complex tasks that a single health care professional cannot handle, and it could also greatly improve the communication structure of the health care workforce [7].

Effective communication among health care professionals is beneficial for patient care, helps foster teamwork among them, and improves their working relationships. Good communication among health care professionals can significantly contribute to clinical effectiveness and job satisfaction [8], whereas poor communication can worsen relationships among medical staff, can negatively affect job satisfaction and turnover, and is a major factor in reducing the well-being of health care providers [3,4,9]. Therefore, clarifying the detailed relationship between the communication structure among medical professionals and the well-being of medical staff in the ICU, which is a stressful workplace with many patients who are critically ill, is desirable; however, comprehensively measuring face-to-face interactions among medical staff is difficult.

### Objective

New wearable technologies have recently been developed to objectively and comprehensively measure face-to-face interactions [10]. Therefore, we aimed to use these techniques to analyze the impact of distributed leadership structure on the amount of face-to-face communication in a prospective observational study. We also measured the well-being of medical staff using scores from the Center for Epidemiological Studies–Depression (CES-D) questionnaire and evaluated the impact of distributed leadership structure on the well-being of medical staff.

## Methods

### Study Design

This was a prospective observational cohort study. The total amount of face-to-face interactions among health care professionals working in the ICU of the tertiary medical center at Mie University and the well-being of the health care professionals were prospectively measured during the observation period.

### Setting

Health care professionals working in the ICU of Mie University Hospital, Japan, participated in this clinical study during each February from 2016 to 2018 (February 2016, February 2017, and February 2018). Specifically, recruitment for the first term began in mid-January 2016, with the research period extending from February 19, 2016, to March 17, 2016. For the second term, participants were recruited in mid-January 2017, and data collection was conducted between February 14, 2017, and March 13, 2017. In the third term, recruitment started in early January 2018, and the research was conducted from February 8, 2018, to March 7, 2018. The ICU is an intensive care center for patients who are critically ill with severe burns, cardiac arrest, septic shock, and trauma. Staff members in the ICU (physicians, nurses, nursing assistants, pharmacists, clinical engineers, receptionists, and secretaries) were included in this study. Participants were recruited 1 month before the beginning of the study and were required to wear wearable sociometric sensor badges (Business Microscope; Hitachi Ltd) on the chest area of their scrubs during work hours only, including breaks, for 4 weeks. These sensors were worn for a duration of 1 month each year and were collected by data analysts at the end of the study period.

### Participants

In this study, all medical staff working in the ICU of Mie University Hospital were included as participants. In 2016, a total of 75 staff members were approached with a written explanation 1 month before the beginning of the study, followed by 93 in 2017 and 85 in 2018. However, each year, consent could not be obtained from 2 nurses, who were therefore excluded from the study. To ensure privacy protection and the integrity of the study, the 2 nurses who did not consent were asked to wear a dummy badge with disabled functions. Therefore, a total of 73 staff members in 2016, a total of 91 in 2017, and a total of 83 in 2018 participated in the study. From all participating health care professionals, data about face-to-face interactions and evaluations of well-being using the CES-D questionnaire were collected. There were no dropouts during the study. Thus, by preserving the completeness of the data, we reduced the potential bias owing to loss to follow-up.

### Assessments

The primary outcome of this study was to collect data about face-to-face interactions among health care professionals over a 1-month period and CES-D scores measured during the same time frame. By analyzing these data, we aimed to elucidate the correlation between face-to-face interactions among health care workers and their sense of well-being. In addition, the study focused on the leadership styles of nurses working in the ICU. In 2016, a traditional leadership model was in place, whereas in 2017 and 2018, the model shifted to a distributed leadership style. This transition allowed for the measurement of changes in communication patterns among health care professionals between 2016 and the subsequent years. In this study, exposure was defined as the distributed leadership structure in 2017 and 2018, compared to the conventional leadership structure in 2016.

### Data Sources

#### Wearable Sensors and Data Collection

Wearable sociometric sensor badges (Business Microscope; Hitachi Ltd) were used, as reported in a feasibility study of an ICU [10] and another study of a corporate call center [11]. The badge worn in the participant’s front pocket automatically and comprehensively collected the data set needed for the social network analysis (SNA) of health care professionals in the ICU. In addition, the badges do not interfere with medical devices, enabling communication and safe collection of behavioral data in real ICU environments. These badges use a 3-axis microelectromechanical accelerometer to capture the physical movements of the wearer and detect personal activities. The badges can also detect face-to-face interactions using 6 infrared, data-related transceivers in front of them [11] and obtain data about who meets with whom, when, and for how long. The wearable sociometric sensor badge captures the physical movements of ICU staff using an accelerometer built into the badge. The greater the frequency with which the acceleration signal exceeds the threshold value per unit of time, the more active the staff member’s body is, indicating the activity level of the staff member. Each staff member’s activity level was evaluated minute by minute; they were classified as being in face-to-face communication if a predetermined threshold was exceeded. On the basis of previous studies’ results, the threshold was set at 2 Hz, a level that could distinguish between active (including conversations with gestures) and quiet (such as keyboard input) actions [11]. Therefore, an active state of face-to-face communication was determined if the threshold value was >2 Hz and the activity lasted for 1 minute. Only face-to-face communications of ≥1 minute were analyzed because a threshold value <1 minute could misinterpret a simple case of staff passing each other in the ICU as active face-to-face communication.

#### SNA (Face-to-Face Interaction Analysis)

##### Overview

SNA was performed using the data set obtained from the wearable sensors, as previously described [11]. It was performed using the UCINET software (Analytic Technologies Inc). The relationship between individuals and their counterparts was evaluated using a centrality measure in the SNA. Furthermore, the following centrality measurement indicators were used.

##### Betweenness Centrality

The betweenness centrality represents a person’s relevance in the sharing of information between people. The ratio of the number of paths that include a person to the total number of paths is known as the person’s betweenness centrality when following the shortest path of combinations across all members. Specifically, a person in the approver role, such as a leader, in a medical context receives a high value [12,13].

##### Degree Centrality

The degree centrality indicates the number of health care professionals to which a person is connected. Specifically, a person who communicates with many health care professionals among the staff members will have a higher value of degree centrality than someone who does not communicate [12,14].

##### Closeness Centrality

The closeness centrality represents the inverse of the mean distance value between 2 people. Particularly, staff members in the ICU who engage in unrestricted communication with everyone without a preference will have a high value of closeness centrality [15].

##### Eigenvector Centrality

People associated with important individuals will have a high eigenvector centrality value. More precisely, people connected to those with high betweenness centrality values show high eigenvector centrality values. In the medical field, well-connected people can spread information quickly; therefore, those with high eigenvector centrality values are considered as important in workplaces with many patients who are critically ill, such as the ICU [16].

#### CES-D Measures of Well-Being

The CES-D questionnaire, which was administered during the third week of the 1-month study, captured the frequency of emotions and behaviors over the past 7 days and was rated on a 4-point scale ranging from 0 (little or none) to 3 (most or all). CES-D comprises 20 items, with a total score that ranges from 0 to 60. High scores indicate great frequency of depressive experiences [17]. Furthermore, a person had to score the lowest (“almost never”) and highest (“almost never”) on all negative (eg, “I felt sad”) and positive (eg, “I enjoyed life”) items, respectively, to score 0 on CES-D. Therefore, implying that these individuals had no depressive experiences would be misleading. Moreover, these individuals also indicated a sense of well-being [18]. Consistent with previous reports, we measured staff well-being using CES-D [19,20].

### Ethical Considerations

This study was conducted in accordance with the ethical standards of the Declaration of Helsinki, and the Human Research Ethics Committee of Mie University approved its protocol (approval 2978). Written informed consent was obtained from all participants. The few staff members who refused participation wore a mock badge, with sensor functions turned off, to preserve anonymity. Furthermore, this clinical trial was registered in the University Hospital Medical Information Network (UMIN) Clinical Trial Registration System (UMIN000037046).

### Sample Size

The purpose of this study was to elucidate the effects of distributed leadership. However, previous studies had not reported about the impact of distributed leadership on CES-D scores, and thus, we estimated an effect size of 0.25 [21]. With a significance level set at .05 and a power of 0.8, the total sample size required over the course of 3 years was calculated to be 159 individuals. Consequently, it was necessary to recruit 53 participants annually. Participation in this study was voluntary, and thus, adequate explanations were provided to all medical personnel working in the ICU to invite participation. Therefore, the number of participants exceeded the required sample size each year, with a total of 73 individuals in 2016, a total of 91 in 2017, and a total of 83 in 2018, thus securing a statistically appropriate sample size for the study.

### Statistical Analysis

**Figure 1:**
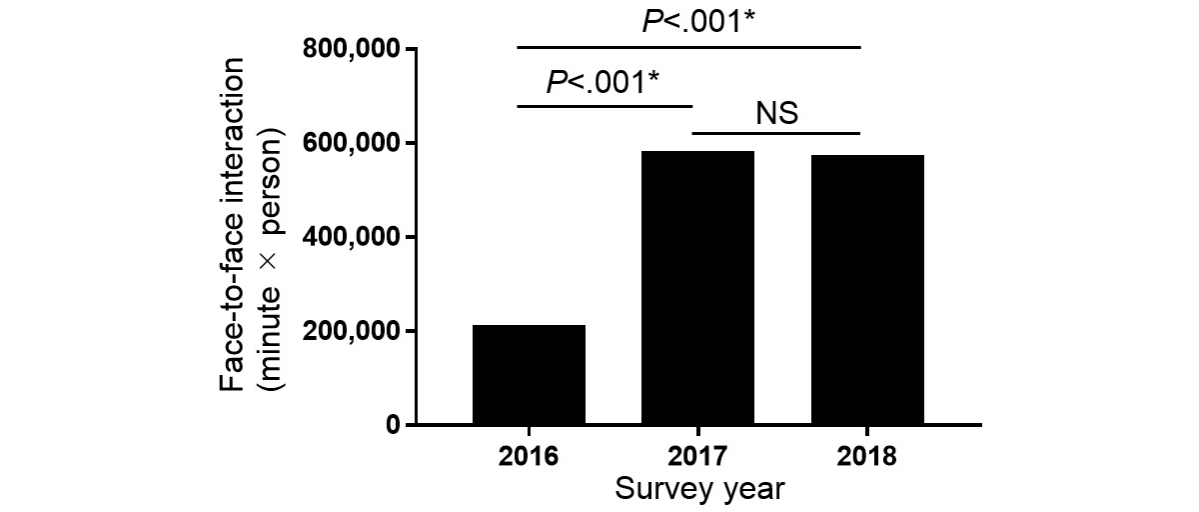
Face-to-face interactions (minute×person) among health care professionals were compared annually. A chi-square test was used for categorical variables. NS: not significantly different. *Statistical significance was set at *P*<.05.

**Figure 2:**
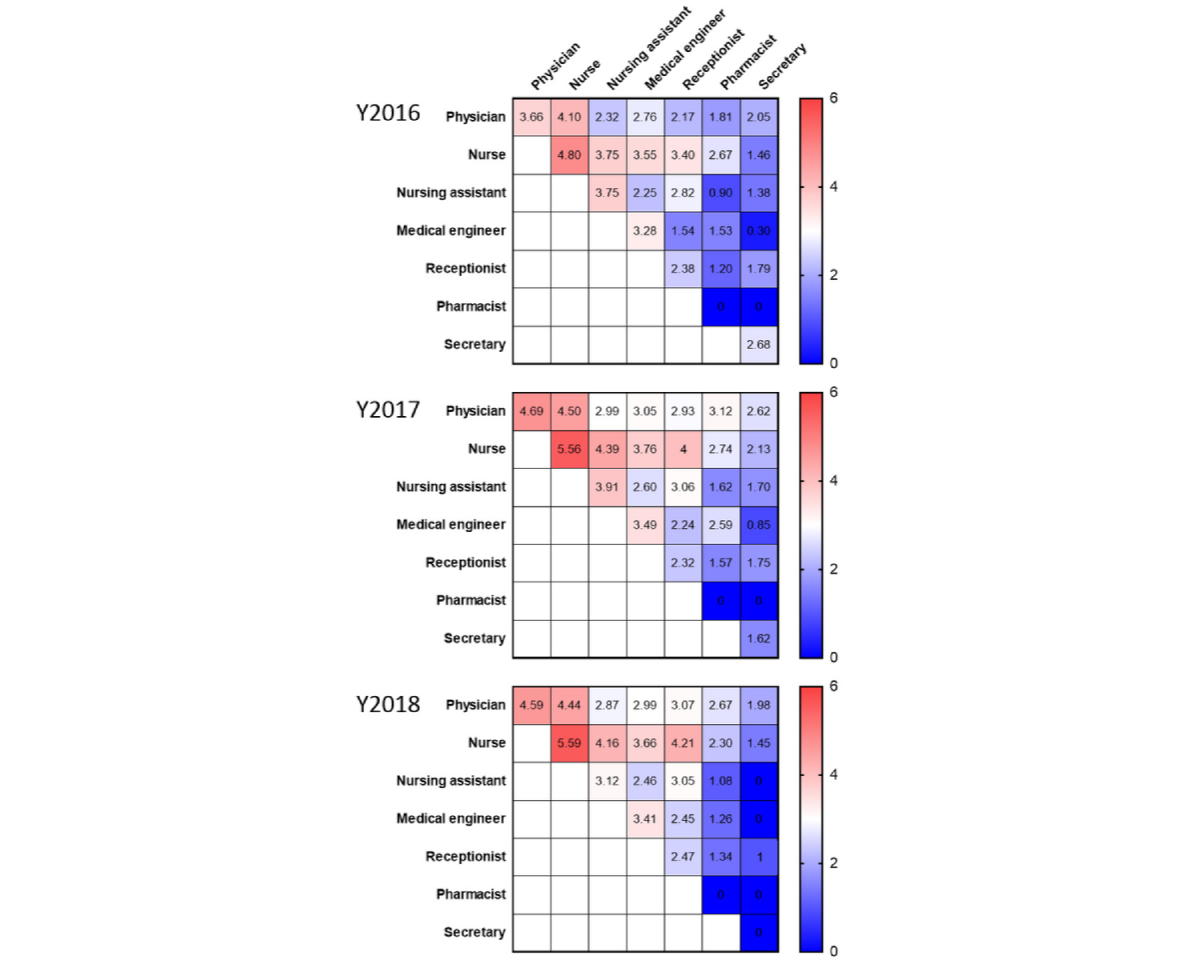
Heat map representation shows the cumulative duration of face-to-face interactions (minute×person) between specific professions. Data are displayed on a logarithmic scale that reflects the duration of the interactions multiplied by the number of people.

Categorical data were compared among study years using chi-square test and reported as frequencies and percentages. Quantitative comparisons of face-to-face interactions among study years, as shown in Figure 1, used the chi-square goodness-of-fit test for comparisons among the 3 groups. While analyzing the face-to-face interactions according to occupation, shown as a heat map in Figure 2, the correlation coefficient was illustrated using Spearman rank correlation coefficient. Heat map representation shows the cumulative duration of face-to-face interactions (minute × person) between specific professions. Statistical analysis was performed using the Kruskal-Wallis test while analyzing non–normally distributed data. Descriptive data were expressed as medians and IQRs. Correlation coefficients (*r*) between CES-D and SNA centrality were expressed using Spearman coefficients. The Shapiro-Wilk test, a hypothesis test that evaluates whether a data set is normally distributed, was used to evaluate normality*.* Statistical significance was set at *P*<.05, and all analyses were performed using IBM SPSS Statistics for Windows (version 23.0; IBM Corp).

## Results

### Demographic Characteristics of All Health Care Providers

Overall, 247 health care professionals participated in the study across the 3 years from 2016 to 2018. Between 2016 and 2018, no significant differences were found in the number of health care professionals, such as physicians, nurses, clinical engineers, pharmacists, nursing assistants, secretaries, and receptionists (all *P*>.05; Table 1). However, the number of experienced leader nurses who could give instructions to other nurses significantly increased to 24 and 20 in 2017 and 2018, respectively, compared with 10 in 2016 (*P*=.046; Table 1). No change was found in the number of formal leaders across the 3 years in each work zone, with 1 physician and 2 nurses. However, the high number of nurses available to perform the leader’s duties increased the number of informal leader nurses and nurses working cooperatively with the formal leader and other medical staff (*P*=.046).

**Table 1:** Demographic characteristics of all health care providers^a^.

Type of occupation	Participants in 2016 (n=73), n (%)	Participants in 2017 (n=91), n (%)	Participants in 2018 (n=83), n (%)	*P* value^b^
Physician	18 (25)	20 (22)	23 (28)	.73
Nurse	38 (52)	53 (58)	45 (54)	.29
Experienced leader nurse	10 (14)	24 (26)^c^	20 (24)^c^	.046^c^
Clinical engineer	7 (10)	9 (10)	9 (11)	.85
Pharmacist	1 (1)	1 (1)	1 (1)	.99
Nursing assistant	5 (7)	4 (4)	2 (2)	.53
Secretary	2 (3)	2 (2)	2 (2)	.99
Receptionist	2 (3)	2 (2)	2 (2)	.99

^a^Chi-square test was used across the 3 years.

^b^Overall *P* value=.37.

^c^*P*<.05 represents statistical significance.

### Total Frequency of Face-to-Face Interactions

All ICU staff worked an average of 160 hours during the 4 weeks of data collection. Overall, 212,872; 583,876; and 573,586 person-minute face-to-face interactions were obtained from a total of 73 staff members in 2016, a total of 91 in 2017, and a total of 83 in 2018, respectively. Face-to-face interactions significantly increased in 2017 and 2018 compared with those in 2016 (*P*<.001; Figure 1).

### Pair Preferences for Face-to-Face Interactions Among ICU Staff Members

Next, we measured the frequency of face-to-face interactions according to occupation and examined which interprofessional communication dominated the frequency of face-to-face interactions in the ICU (Figure 2). First, we compared the total duration of face-to-face interactions per month according to occupation and by year. The duration of face-to-face interactions involving either physicians or nurses was longer than that of other occupations across the 3 years (2016-2018; Figure 2). In addition, across the 3 years, nurses had the highest frequency of face-to-face interactions with other nurses, and their duration of face-to-face interactions was longer than that among other professionals. Nurses also interacted actively and frequently with other professionals (Figure 2). Physicians communicated most frequently with nurses in 2016 during the 3-year measurement, whereas the communication frequency among physicians increased in 2017 and 2018. Therefore, these data indicate that nurses play a central role in face-to-face interactions in the ICU.

### Comparison of SNA Centrality Across the 3 Years

**Figure 3:**
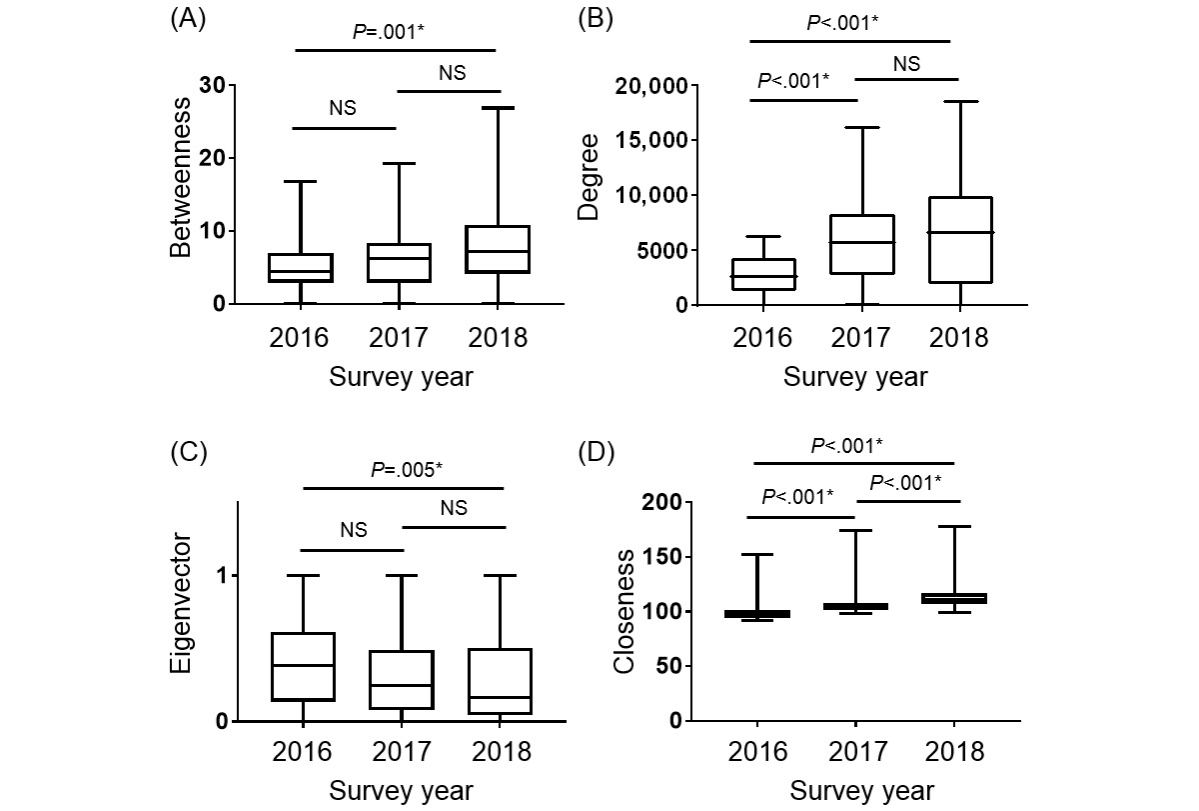
The face-to-face interaction data obtained from the wearable sensors were used to analyze the centrality of health care professionals and were compared with 3 years of data. Overall, 4 types of centralities are illustrated: (A) betweenness, (B) degree, (C) eigenvector, and (D) closeness centralities. Descriptive data are summarized as medians and IQRs. NS: not significantly different. *Statistical significance was set at *P*<.05.

We conducted a 3-year SNA of health care professionals to measure the centrality of SNA. Specifically, we measured 4 centralities as follows: the betweenness, degree, eigenvector, and closeness centralities. The high betweenness centrality for approvers in an organization (ie, leader physicians and nurses) significantly increased in 2018 compared with that in 2016 (*P*=.001; Figure 3A). Notably, this could be attributed to a significant increase in nurses’ experience as leader nurses since 2017 (Table 1), the emergence of informal leaders to assist leaders in the organization, and more staff members communicating collaboratively with other staff members. The degree centrality, which represents the connection with many health care professionals, significantly increased in 2017 and 2018 compared with that in 2016 (*P*<.001; Figure 3B), and this could be because of the facilitated communication among staff members. Furthermore, the eigenvector centrality, which is high among staff members connected to many health care professionals, significantly decreased in 2018 compared with that in 2016 (*P*=.005; Figure 3C), indicating that more individuals played a role in rapidly disseminating information in the ICU in 2016 than in 2018. Specifically, information was transmitted by few leaders but received by many staff members, suggesting that leadership duties were undistributed. More interestingly, the closeness centrality, which is high for staff members who can communicate with everyone without any preference, significantly increased in 2017 and 2018 compared with that in 2016 (*P*<.001; Figure 3D). This result strongly supports the notion that informal leaders were created to assist those in the organization after 2017, when a distributed leadership structure was adopted, and that more staff members communicated collaboratively with others.

### Changes in Well-Being Over the 3 Consecutive Years

**Figure 4:**
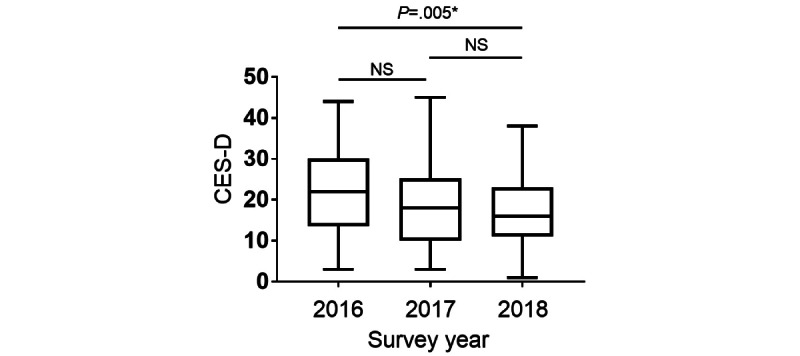
The Center for Epidemiological Studies–Depression (CES-D) questionnaire was administered during the third week of the 1-month study. Descriptive data are summarized as medians and IQRs. NS: not significantly different. *Statistical significance was set at *P*<.05.

All participants (247/247, 100%) were asked to complete the CES-D questionnaire during the third week of the study to measure their well-being (Figure 4). High CES-D scores were positively correlated with the degree of depression, whereas the lower the CES-D scores, the happier the health care provider [19,20]. The median CES-D scores were 22 (IQR 14-30), 17 (IQR 10-25), and 16 (IQR 11-23) in 2016, 2017, and 2018, respectively. CES-D scores were significantly decreased in 2018 compared with those in 2016, suggesting an increase in the well-being of health care professionals in 2018 (*P*=.005). Generally, a score ≥16 indicates depression; therefore, these results show that although health care professionals were exposed to excessive stress, the situation gradually improved over the 3 consecutive years [17].

### Correlations Between the Well-Being of Health Care Professionals and SNA Centrality

**Figure 5:**
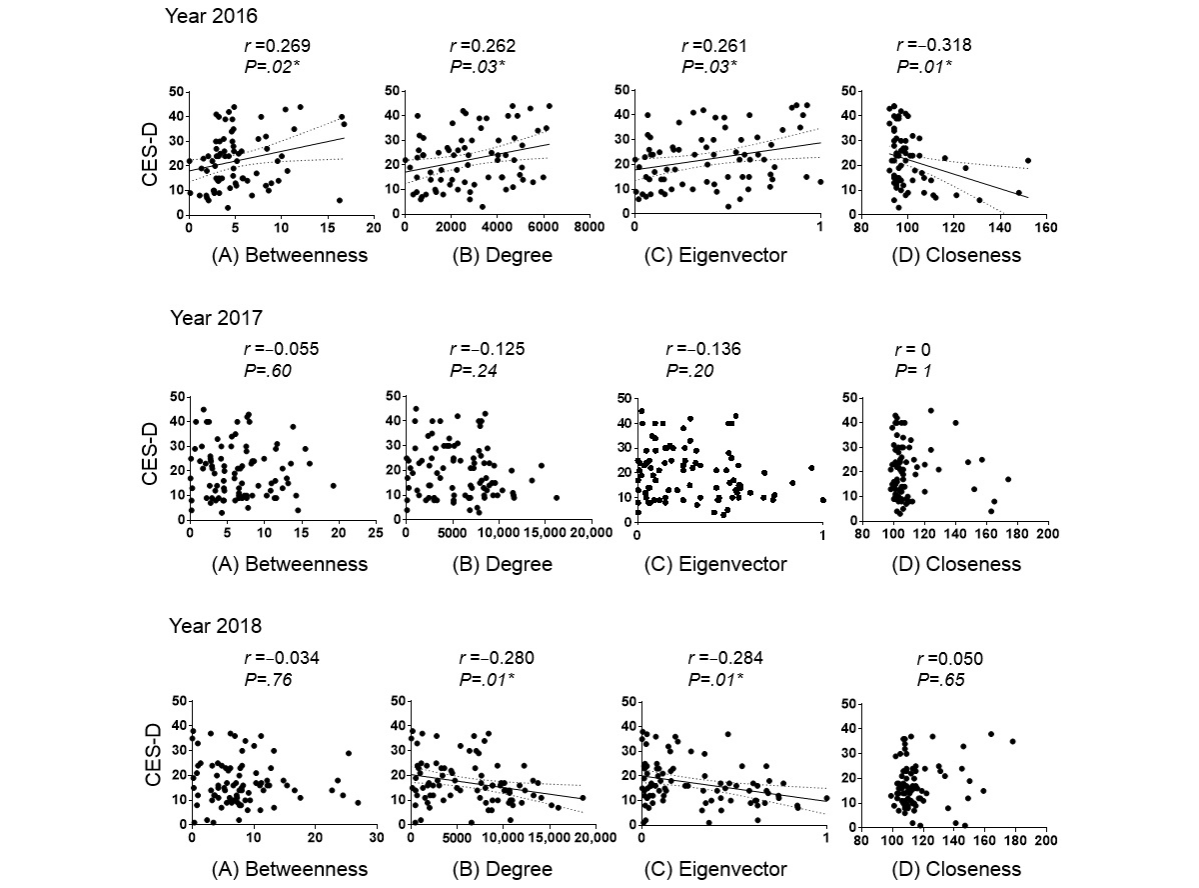
The correlation between the Center for Epidemiological Studies–Depression (CES-D) scores and the 4 centralities, which include (A) betweenness, (B) degree, (C) eigenvector, and (D) closeness centralities, is illustrated yearly. The correlation coefficients (r) between the CES-D scores and social network analysis centrality are expressed using Spearman coefficients. *Statistical significance was set at *P*<.05.

Next, we analyzed the correlation between the well-being of all staff members and the centrality of the SNA (Figure 5). In 2016, the betweenness centrality increased and was positively correlated with health care professionals with high CES-D values (ie, those with low happiness and high depression levels; *r*=0.269; *P*=.02). The CES-D scores were also positively correlated with health care professionals with high degree centrality values (*r*=0.262; *P*=.03) and positively correlated with the eigenvector centrality (*r*=0.261; *P*=.03). These results strongly suggest that the leadership duties of some staff members placed excessive psychological burden and workload on others in 2016. Interestingly, the CES-D scores were also negatively correlated with the closeness centrality in 2016 (*r*=−0.318; *P*=.006). In addition, this result shows that people who could communicate with everyone in the organization without any preference had high levels of well-being, suggesting that effective communication positively influenced the well-being of staff members. Although no correlation was found between SNA centrality and CES-D scores in 2017 (Figure 5), the degree (*r*=−0.280; *P*=.01) and eigenvector (*r*=−0.284; *P*=.009) centralities were negatively correlated with CES-D scores in 2018. Moreover, opposite results were obtained in 2016 and 2018. Therefore, this result shows that staff members connected to many health care professionals have high level of well-being.

### Demographic Characteristics of Health Care Providers Over the 3 Consecutive Years

We further conducted a subgroup analysis to ascertain whether similar results would be found among the staff members who participated in this study over the 3 consecutive years. Overall, 42 health care professionals participated in the study over the 3 consecutive years, of whom 7 (17%) were physicians, 26 (62%) were nurses, 4 (10%) were clinical engineers, 1 (2%) was a pharmacist, 2 (5%) were nursing assistants, and 2 (5%) were receptionists (Table 2). Among the 42 health care professionals, 6 (14%) nurses were available for leadership duties in 2016, significantly increasing to 19 (45%) in 2017 and 20 (48%) and 2018 (*P*=.02; Table 2).

**Table 2:** Demographic characteristics of participants over the 3 consecutive years^a^.

Type of occupation	Participants in 2016 (n=42), n (%)	Participants in 2017 (n=42), n (%)	Participants in 2018 (n=42), n (%)	*P* value^b^
Physician	7 (17)	7 (17)	7 (17)	.99
Nurse	26 (62)	26 (62)	26 (62)	.99
Experienced leader nurse	6 (14)	19 (45)^c^	20 (48)^c^	.02^c^
Clinical engineer	4 (10)	4 (10)	4 (10)	.99
Pharmacist	1 (2)	1 (2)	1 (2)	.99
Nursing assistant	2 (5)	2 (5)	2 (5)	.99
Secretary	0 (0)	0 (0)	0 (0)	.99
Receptionist	2 (5)	2 (5)	2 (5)	.99

^a^Chi-square test was used across the 3 years.

^b^Overall *P* value=.99.

^c^*P*<.05 represents statistical significance.

### Comparison of SNA Centrality Among Health Care Professionals Who Participated in the Study Over the 3 Consecutive Years

**Figure 6:**
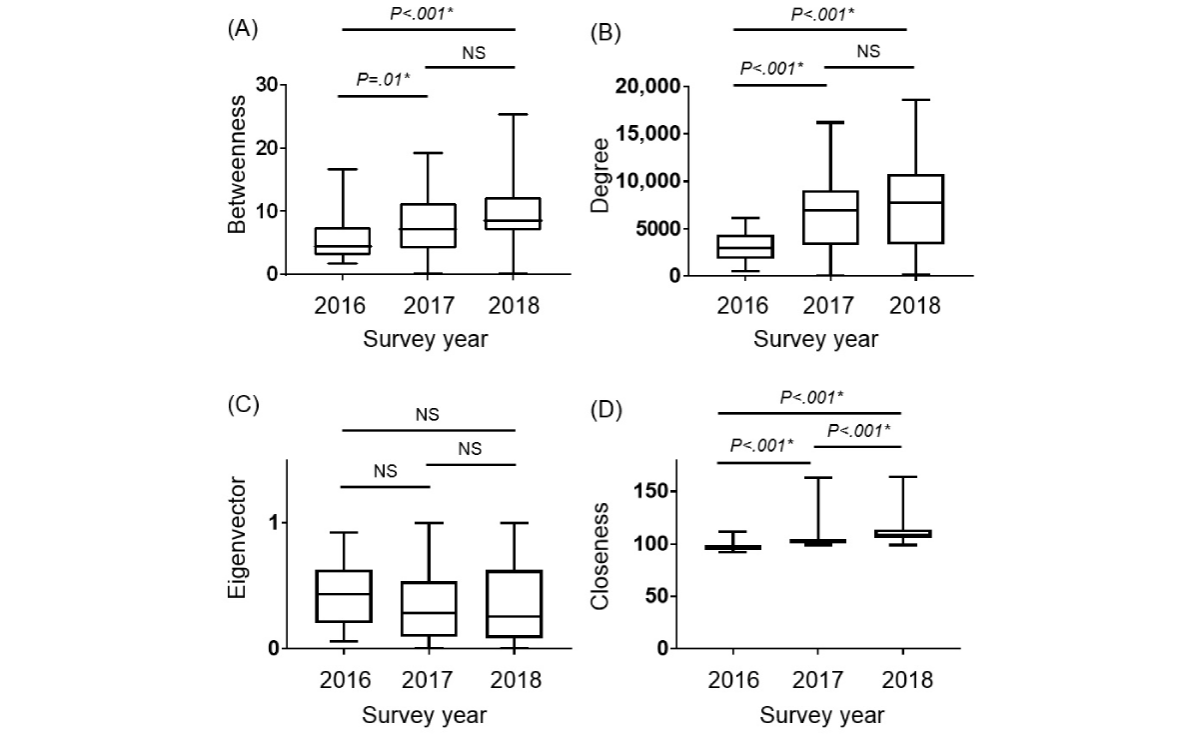
The face-to-face interaction data obtained from the wearable sensors were used to analyze the centrality of health care professionals and were compared among the 3 years of data. Overall, 4 types of centralities are illustrated as follows: (A) betweenness, (B) degree, (C) eigenvector, and (D) closeness centralities. Descriptive data are summarized as medians and IQRs. NS: not significantly different. *Statistical significance was set at *P*<.05.

In the subgroup analysis, the betweenness centrality was significantly increased in 2017 (*P*=.01) and 2018 (*P*<.001) compared with that in 2016 for the 42 health care professionals who participated in the study over the 3 consecutive years (Figure 6A). The degree centrality was also significantly increased in 2017 and 2018 compared with that in 2016 (*P*<.001; Figure 6B), whereas the eigenvector centrality did not differ across the 3 years of measurement (Figure 6C). The closeness centrality was significantly increased in 2017 and 2018 compared with that in 2016 (*P*<.001) and significantly increased in 2017 compared with that in 2018 (*P*<.001; Figure 6D). Subgroup analyses of the 42 health care professionals who participated in the study over the 3 consecutive years also showed increases in the betweenness, degree, and closeness centralities, similar to the analysis results of all health care professionals.

### Trends in the Well-Being of Health Care Professionals Who Participated in the Study Over the 3 Consecutive Years

**Figure 7:**
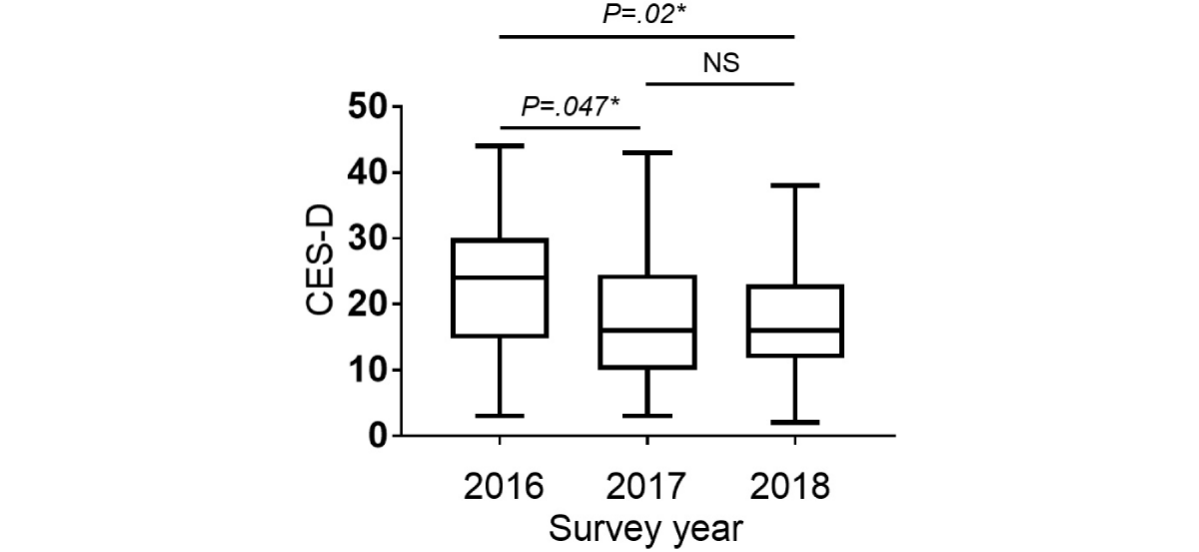
The Center for Epidemiological Studies–Depression (CES-D) questionnaire was administered during the third week of the 1-month study. Descriptive data are summarized as medians and IQRs. NS: not significantly different. *Statistical significance was set at *P*<.05.

The median CES-D scores for the 42 health care professionals who participated in the study over the 3 consecutive years were 24 (IQR 15-30), 16 (IQR 10-24), and 16 (IQR 12-23) in 2016, 2017, and 2018, respectively. Furthermore, the CES-D scores decreased in 2017 (*P*=.047) and 2018 (*P*=.02) compared with that in 2016 (Figure 7), whereas well-being significantly increased in the subgroup analyses.

### Correlations Between Well-Being and SNA Centrality for Health Care Professionals Who Participated Over the 3 Consecutive Years

**Figure 8:**
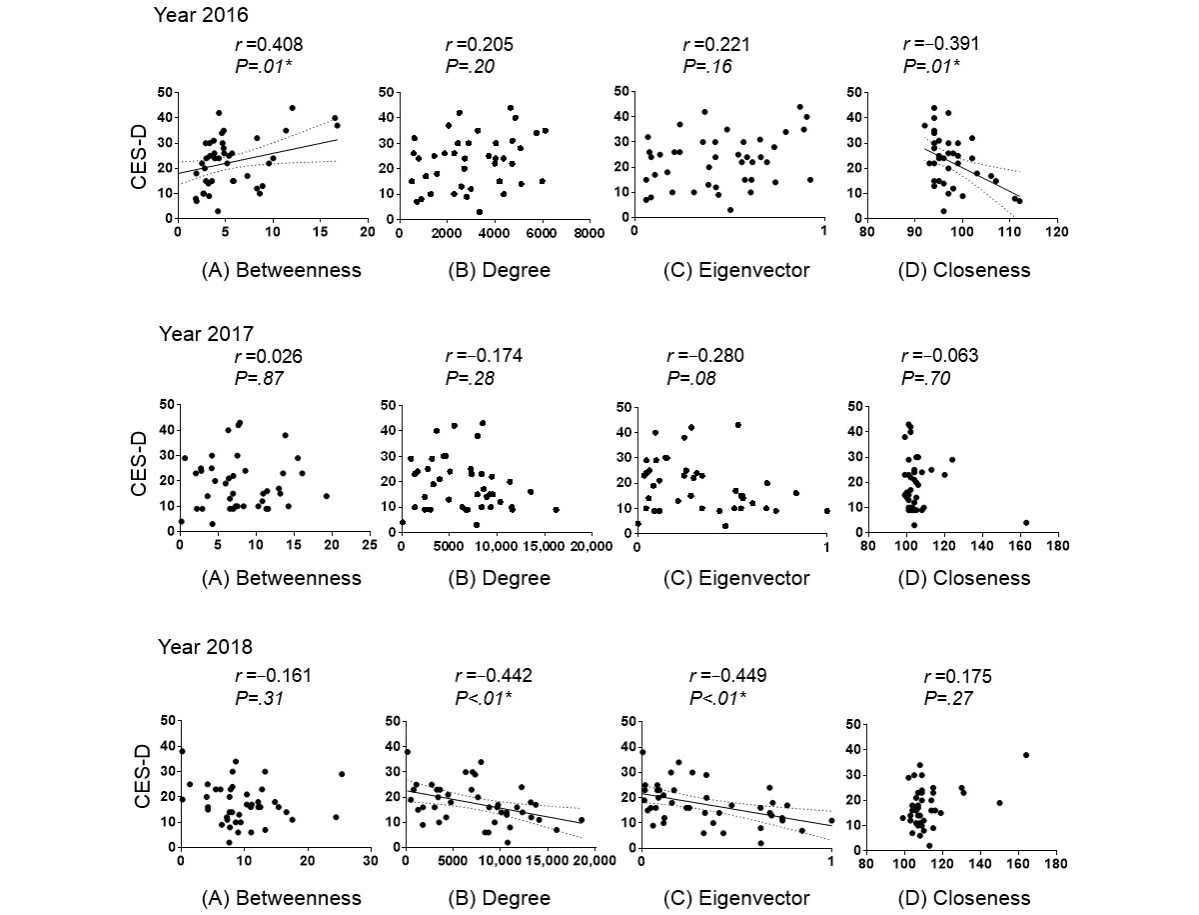
The correlation between the Center for Epidemiological Studies–Depression (CES-D) scores and the 4 centralities, which include (A) betweenness, (B) degree, (C) eigenvector, and (D) closeness centralities, is illustrated yearly. The correlation coefficients (r) between the CES-D scores and social network analysis centrality are expressed using Spearman coefficients. *Statistical significance was set at *P*<.05.

Next, we analyzed the correlation between well-being and SNA centrality for the 42 staff members who participated over the 3 consecutive years (Figure 8). In 2016, the betweenness centrality was positively correlated with CES-D scores (*r*=0.408; *P*=.007), whereas the closeness centrality was negatively correlated with CES-D scores (*r*=−0.391; *P*=.01). No correlation was found between CES-D scores and SNA centrality in 2017. In 2018, CES-D scores were negatively correlated with the eigenvector (*r*=−0.449; *P*=.003) and degree (*r*=−0.442; *P*=.003) centralities. Therefore, this result indicates that staff members who are connected to many health care professionals have high levels of well-being. In addition, staff members connected to health care professionals with high values of betweenness centrality (ie, health care professionals with high eigenvector centrality), such as leader nurses, showed high levels of well-being.

## Discussion

### Principal Findings

In this study, we found that nurses play a central role in face-to-face interactions in the ICU (Figures 1 and 2). Furthermore, since 2017, a distributed leadership structure has been implemented in the ICU, with the number of approvers (ie, leader physicians and nurses) and informal leaders in the organization significantly increasing in 2018 compared with that in 2016. Therefore, communication among staff members was facilitated, and communication between many health care professionals and others was improved. Interestingly, the activation of communication in the ICU also increased the well-being of health care professionals, particularly the well-being of medical staff, which increased with those who extensively communicated with the surrounding staff.

Distributed leadership was introduced to the ICU nursing staff members during the second and subsequent years of the study, dramatically altering the centrality of the communication structure among medical staff. The betweenness, degree, and closeness centralities increased after 2017 compared with those in 2016. This result supports the idea that a distributed leadership structure creates informal leaders who collaborate and facilitate interprofessional communication in the organization (Figure 3). Interestingly, the level of depression among ICU staff members improved as cooperation increased in the organization (Figure 4). Owing to the high number of patients who are critically ill in the ICU, a few staff members perform leadership duties, which decreases the well-being of some staff members and the organization’s overall performance. Therefore, introducing a distributed leadership structure to overcome this situation and create effective collaboration could improve the ICU work environment. Similar results were shown in a study that conducted an SNA with similar wearable technology for employees at a company outside the health care industry [22]. In a previous study, the betweenness centrality in the SNA was highly negatively correlated with satisfaction with group interactions [22]. Therefore, overloading employees, such as with leadership duties, may reduce the well-being of some individuals, consequently reducing the satisfaction and well-being of the organization.

The comprehensive measurement of face-to-face interactions among health care professionals requires using new technologies rather than traditional, survey-like methods. Therefore, we addressed this issue using a wearable social measurement sensor badge (Hitachi Ltd) to measure face-to-face interactions among health care professionals in the ICU. Furthermore, SNA using the data set obtained from the wearable sensor revealed the structure of interprofessional interactions in the ICU and showed that nurses play a pivotal role in the interaction network (Figures 1 and 2). Similar sociometric wearable sensor technologies have also been used to quantify the dynamics of health care professionals’ behavior in complex and unpredictable health care environments. For example, it has been used in studies of the interactions among health care professionals, patients, and caregivers in a general children’s hospital ward in Italy [23]; among nurses in a surgical ICU at a university hospital in the United States [24]; and in the emergency department of a university hospital in the United States [25]. Therefore, the ability to monitor face-to-face interactions in health care settings using wearable sensors has been supported by several independent studies, including ours [10].

SNA is a research approach in the social sciences that focuses on analyzing interactions between individuals and social groups to explain social patterns of behavior and interest [26,27]. SNA studies in health care have examined various topics, such as patient satisfaction with the social support received by patients with chronic pain [28], studies of professional network structure [29], and health care provider job satisfaction and leadership; it has also been used to demonstrate various outcomes [30,31]. However, SNA remains underused in understanding professional communication and performance among health care providers owing to the difficulty in accurately measuring communication among them [32]. We accurately measured face-to-face interactions using a wearable sensor in this study, which allowed us to use SNA to prospectively analyze accurate face-to-face interactions among health care professionals according to occupation and over 3 consecutive years.

Distributed leadership or collaborative leadership is a novel theory in this field. Supporting and building relationships with health care professionals increase the likelihood that they will be positively influenced and motivated to work toward their goals. Furthermore, the emergence of supportive leaders who empathize on a personal level enables staff to be happy and more satisfied with their work [5]. The distributed leadership introduced in this study may have developed cooperativeness and enabled the organization to support informal leaders in communicating information to colleagues and related organizations, thus enabling all health care professionals to make well-informed decisions. In addition, informal leaders participated in the leadership process and were actively involved in identifying and communicating the needs of health care professionals, which may have altered the communication structure of health care professionals, reduced the psychological and physical burden on formal leaders, and increased the well-being of organizations.

Improved teamwork and communication are among the most important factors for health care professionals to improve their clinical effectiveness and job satisfaction [8]. Therefore, activating communication among health care professionals may improve patient life outcomes in the ICU [33-35]. As we found in this study, the introduction of distributed leadership may improve patient life outcomes by changing the structure of SNA among ICU staff members and increasing their well-being. However, further prospective clinical studies are needed to confirm this hypothesis.

In this study, the CES-D scale was used to measure well-being. Unfortunately, the CES-D scale does not measure well-being comprehensively, as it assesses the continuum from happiness to depression [20]. However, the CES-D scale has established reliability and validity in a wide range of research for the assessment of the presence and severity of depressive symptoms, and it is deemed suitable for capturing the negative aspects of mental health among health care workers, which is why it was adopted for this study. However, for a more extensive evaluation of well-being, it would be desirable to use additional measures that assess subjective happiness in conjunction with the CES-D scale. This approach would enable the assessment of not only the presence of depression but also positive aspects such as sense of happiness and quality of life. Future studies should aim for a more holistic understanding of well-being by using composite measures that multifacetedly evaluate psychological well-being [36].

The measurement of face-to-face interactions using wearable sensors revealed that nurses were central to ICU communication. Furthermore, in stressful workplaces, such as the ICU, the well-being of medical staff members in leadership positions is reduced owing to excessive workload and psychological burden.

However, distributed leadership can increase organizational well-being by changing the communication structure of health care professionals in the ICU and creating a cooperative environment in the workplace.

### Comparison With Previous Studies

One of the impediments to the implementation of SNA in the field of health care research is the lack of effective techniques for objective and comprehensive data acquisition [31]. Currently, data sets for SNA are commonly obtained through manual acquisition of questionnaires, observations, and electronic medical and administrative records [37,38]. However, objectively, comprehensively, and continuously measuring social network connections among health care professionals with such manual data acquisition is difficult. Therefore, in this study, we overcame these difficulties by using wearable sensors to objectively and comprehensively collect data about the duration of face-to-face communication among health care professionals in the ICU. To the best of our knowledge, this is the first study to reveal the impact of distributed leadership on the centrality of health care worker communication networks and the well-being of ICU health care professionals. Therefore, comparing our findings with those of previous studies is not practical, given the novelty of our approach.

### Limitations

This study had some limitations. First, this was an observational study; therefore, it could reveal a causal relationship between a distributed leadership structure and the well-being of ICU staff members. However, as nurses are central to the ICU communication structure, it is reasonable to assume that changes in the leadership structure of nurses and their colleagues would influence the level of organizational well-being. Furthermore, a previous study showed that the key elements for effective physician-nurse teamwork are the quality of collaboration, coordination, shared mental models, communication structure, and leadership structure [39]. Leadership structure largely influences ICU communication structure and teamwork [39]. Therefore, an improved ICU work environment resulting from implementing a distributed leadership structure can improve the well-being of health care professionals in the ICU. For example, implementing distributed leadership may effectively prevent burnout, which many ICU staff experience.

Second, this study did not measure communication other than face-to-face interactions, as measured using wearable badges. For example, digital web-based information exchange and communication via email and mobile phones, among others, which have rapidly become mainstream in today’s society, were not included in the information obtained from wearable sensors. However, the most important human interactions in the ICU still occur offline, and face-to-face interactions are essential for building organizational trust [40]. Therefore, this study focused on offline, face-to-face interactions.

### Conclusions

Our study revealed a relationship between the ICU communication structure and the well-being of health care professionals. Specifically, the well-being of health care professionals with high degree centrality values, who communicate better with other health care professionals, was increased, suggesting that the promotion of interdisciplinary medical care can stimulate communication within the ICU and increase the level of well-being in the workplace. In addition, the level of well-being of health care professionals with high betweenness centrality, such as leader physicians and nurses, was low. These results suggest that in stressful workplaces, including ICUs, the excessive emotional burden placed on some leader physicians and nurses weighs on them, resulting in poor organizational performance. However, a possible solution to this problem is introducing a distributed leadership structure, where leadership duties can be shared among multiple staff members to reduce the burden on 1 person.

In summary, this study shows that increasing the number of experienced leader nurses and promoting a distributed leadership structure can enhance the well-being of health care professionals in ICUs.

## Abbreviations

CES-D: Center for Epidemiological Studies–Depression
HCP: health care professional
ICU: intensive care unit
SNA: social network analysis
UMIN: University Hospital Medical Information Network

## Data Availability

The data sets analyzed during this study are available from the corresponding author upon reasonable request.
